# SG-APSIC1107: Surgical-site infection in Hung Vuong Hospital, a gynecology and obstetrics tertiary-care hospital in Ho Chi Minh City, Vietnam

**DOI:** 10.1017/ash.2023.90

**Published:** 2023-03-16

**Authors:** Anh Dinh, Phan Thi Hang, To Gia Kien, Tran Thi Thuy Hang, Ngo My Nhung, Ngo Thi Thanh Tham

**Affiliations:** Hung Vuong Hospital, Ho Chi Minh, Vietnam; Hung Vuong Hospital, Ho Chi Minh, Vietnam; University of Medicine and Pharmacy, Ho Chi Minh, Vietnam; Hung Vuong Hospital, Ho Chi Minh, Vietnam; Hung Vuong Hospital, Ho Chi Minh, Vietnam; Hung Vuong Hospital, Ho Chi Minh, Vietnam

## Abstract

**Objectives:** Surgical site infection (SSI) is the most common healthcare-associated infection (HAI) in our gynecology and obstetrics hospital. SSI among patients following gynecological and obstetrical surgery not only results in increased morbidity but also has far-reaching implications. Thus, this study was conducted to determine the incidence, risk factors, and bacterial pathogens related to SSI. **Methods:** We conducted this retrospective study based on medical records from January 2019 to December 2020 at Hung Vuong Hospital. **Results:** Of 51,466 patients undergoing surgery, 581 patients (1.34%) developed an SSI after cesarean section and 145 patients (1.77%) developed an SSI after gynecological surgery. A multivariate logistic regression analysis identified the following risk factors among patients who underwent cesarean section: age (OR, 1.02; 95% CI, 1.01–1.04), emergency cesarean section (OR, 1.62; 95% CI, 1.36–1.93), operation time >60 minutes (OR, 2.04; 95% CI, 1.48–2.80), surgery during the night shift (OR, 1.29; 95% CI, 1.08–1.54), and prolonged hospital stay ≥2 days (OR, 1.51; 95% CI, 1.21–1.89). SSI risk factors for patients following gynecological surgery included age (OR, 1.03; 95% CI, 1.02–1.05), contaminated wound (OR, 3.44; 95% CI, 1.56–7.57), dirty wound (OR, 3.61; 95% CI, 1.44–9.05), vertical abdominal incision (OR, 2.49; 95% CI, 1.65–3.77), and duration of surgery >180 minutes (OR, 2.02; 95% CI, 1.24–3.29). *Staphylococcus aureus* was the most commonly identified SSI pathogen following cesarean section (49.56%), and *Escherichia coli* was isolated in 44.93% of SSIs among patients undergoing gynecological surgery. **Conclusions:** SSI interventions should target this high-risk group. Based on microbiology culture and susceptibility results isolated from SSI cases, novel antibiotic therapies are needed to treat SSIs.

